# Study of the Regulatory Role of *N*-Acyl Homoserine Lactones Mediated Quorum Sensing in the Biological Activity of *Burkholderia gladioli* pv. *agaricicola* Causing Soft Rot of *Agaricus* spp.

**DOI:** 10.3389/fmicb.2019.02695

**Published:** 2019-11-29

**Authors:** Hazem S. Elshafie, Giulia Devescovi, Vittorio Venturi, Ippolito Camele, Sabino A. Bufo

**Affiliations:** ^1^School of Agricultural, Forestry, Food and Environmental Sciences, University of Basilicata, Potenza, Italy; ^2^Bacteriology Group, International Centre for Genetic Engineering and Biotechnology, Trieste, Italy; ^3^Department of Science, University of Basilicata, Potenza, Italy; ^4^Department of Geography, Environmental Management and Energy Studies, University of Johannesburg, Johannesburg, South Africa

**Keywords:** phytopathogenic bacteria, antagonistic activity, *Agaricus bisporus*, secondary metabolites, quorum sensing, HPLC

## Abstract

Many *Burkholderia* spp. produce *in vitro* secondary metabolites with relevant biological activities and potential practical applications. *Burkholderia gladioli* pv. *agaricicola* (*Bga*) possess promising biological activities regulated by *N*-Acyl homoserine lactones (*N*.AHLs) based quorum sensing (QS) mechanism. In the current study, *N*.AHLs-deficient (*ICMP11096glad-*I) and *N*.AHLs-complemented (*ICMP11096glad-*IR) mutants were constructed in which the gene coding for AHL synthase was inactivated by allelic exchange in *glad* I mutant strain. The aims of this research were to (i) assess the antagonistic activity of the wild type (WT) and the *glad-*I mutant of *Bga* against *Bacillus megaterium* (G+ve) and *Escherichia coli* (G−ve), (ii) screen their hydrolytic enzymes and hemolytic substances, (iii) monitor the pathogenic effect against *Agaricus bisporus*, and finally (iv) analyze the bioactive secondary metabolites produced by WT and mutant strain using high performance liquid chromatography (HPLC). Results showed that *N.*AHLs-deficient mutant exhibited high reduction of antagonistic activity against the tested microorganisms and notable reduction of chitinolytic, proteolytic and glucanolytic activities and complete absence of hemolytic activity, and the *glad-*IR complemented mutant was able to regain the major part of these activities. Furthermore, *N*.AHLs-deficient mutant strain was unable to degrade flesh cubes pseudo-tissues of *A. bisporus*. On the other hand, the virulence effect of complemented mutant was like to the parental WT strain. HPLC analysis revealed that some of the single components produced by WT strain were absent in *N.*AHLs-deficient mutant and others were highly reduced. The out-findings of the current research gave a spot into the regulatory role of *N*.AHLs and QS phenomenon in the biological activity of *Bga* bacterium.

## Introduction

Many bacteria regulate their biological activities and interaction with surrounding conditions through a particular mechanism called quorum sensing (QS) (Steindler and Venturi, 2007). This mechanism enables the bacterial cells to communicate to each other by responding to different signal molecules (Withers et al., 2001).

Quorum sensing mechanism in bacteria is classified into three main classes: LuxI/LuxR in Gram-negative (G−ve) bacteria; oligopeptide-two-component G+ve bacteria; and *luxS*-encoded autoinducer 2 (AI-2) in both G−ve and G+ve bacteria (Withers et al., 2001; Li and Tian, 2012). The signal molecules are also divided into two main groups: *N*-Acyl homoserine lactones (*N*.AHLs) in G−ve and small peptides in G+ve bacteria (Davey and O’Toole, 2000; Costerton et al., 2003). QS regulates the production of virulence factors, bioactive metabolites, symbiosis, biofilm formation and motility for colony escape (Kumari et al., 2006; Duerkrop et al., 2007; Zhang et al., 2012). *N.*AHLs mediated QS have already been reported as regulators for several primary and secondary metabolites in some G−ve bacteria belonging to *Pseudomonas* and *Burkholderia* species and to *Agrobacterium* genus (Zhou et al., 2003; Dong and Zhang, 2005).

*Burkholderia gladioli* Yabuuchi is a widespread G−ve bacteria in large variety of sources, including soil, water, and vegetation (Yabuuchi et al., 1992; Coenye and Vandamme, 2003; Stoyanova et al., 2007). Originally *B. gladioli* wa*s* considered a causal agent of cavity disease of mushroom (Chowdhury and Heinemann, 2006). It was also considered a plant growth promoting rhizobacterium (Karakurt and Aslantas, 2010; Elshafie et al., 2017b). *B. gladioli* is also considered an important human pathogen causing fibrocystic lung disease (Wilsher et al., 1997).

*Burkholderia gladioli* pv. *agaricicola* (*Bga*) is one of the most serious pathogens in the mushroom industry (Gill, 1995). It causes soft rot on a number of commercially important mushrooms such as *Lentinula edodes*, *Pleurotus ostreatus*, *Flammulina velutipes*, *Pholiota nameko*, *Hypsizygus marmoreus*, and *Grifola frondosa* in Japan and different cultivated *Agaricus* species in New Zealand and Europe (Chowdhury and Heinemann, 2006).

Recent investigations have shown that *Bga* produces *N.*AHLs (Prashanth et al., 2011) but their regulatory role in virulence and other phenotype traits and biological activity are not yet known (Andolfi et al., 2008; Cimmino et al., 2008; Elshafie et al., 2012). In particular, Elshafie et al. (2017a) have studied the *in vitro* antimicrobial effects of four wild type (WT) strains of *Bga* and their hemolytic, enzymatic and virulence activities, and results showed that all WT strains inhibited *Bacillus megaterium* and *E. coli* growth. Results showed also that *Bga* ICMP11096 showed the highest antimicrobial activity toward both target microorganisms, was able to haemolyze erythrocytes cell membrane, and produces the hydrolytic enzymes (chitinase, glucanase, and protease). Moreover, the virulence effect of the four studied strains have been verified against *Agaricus bisporus* tissues (Elshafie et al., 2017a).

The main objective of the current research was to construct *N*.AHLs-deficient (*ICMP11096glad-*I) and *N*.AHLs-complemented (*ICMP11096glad-*IR) mutants of *Bga* ICMP11096 and to study their antimicrobial, hydrolytic, hemolytic, and virulence activities compared to WT strain and to analyze also the produced bioactive fractions of the WT and *ICMP11096glad*-I mutant using high performance liquid chromatography (HPLC).

## Materials and Methods

### Bacterial Strains

The WT strain of *Bga* ICMP11096 has been described in detail in previous works as reported by Elshafie et al. (2017a), and was maintained as lyophils at 4°C, and later on subcultures were obtained on the King B medium (KB) for 48 h at 25°C. Modified *N*.AHLs-deficient mutant of the WT strain (ICMP11096*glad-*I) and the complemented mutant (ICMP11096*glad-*IR) were prepared at the International Centre for Genetic Engineering and Biotechnology (ICGEB), Trieste, Italy. *Chromobacterium violaceum* CV026 sensor was grown in Luria-Bertani medium (LB) at 30°C. The target microorganisms *Bacillus megaterium* ITM100 (*B. megaterium*) and *Escherichia coli* ITM103 (*E. coli*) were previously identified and stored as pure freeze-dried cultures at −20°C in the collection present at the School of Agricultural, Forestry, Food and Environmental Sciences (SAFE), University of Basilicata, Potenza, Italy. Conjugations in *B. gladioli* strains were performed by triparental mating using *E. coli* DH5 (pRK2013) as helper and incubated at 30°C for 12 h. Transconjugants were selected on KB medium containing the appropriate antibiotics. The plasmids in this study are listed in Table 1.

**TABLE 1:** Bacterial strains and plasmids used in this study.

**Strain or plasmid**	**Description**	**Source or reference**
*B. gladioli* pv *agaricicola* strains		
ICMP 11096		
ICMP 11096glad-I	*gladI*:Km of *B. gladioli* 11096	This study
ICMP 11096glad-IR	ICMP 11096gladI complemented with pBBgladIR	This study
CV026	*C. violaceum* AHLs sensor strain; double transposon mutant of ATCC31532, violacein and AHL negative	McClean et al., 1997
pGEM2T easy	Cloning vector Amp^R^	Promega
pLAFR3	Broad-host-range cloning vector, IncP1, Tc^R^	Staskawicz et al., 1987
pRK2013	Km^R^ Tra + Mob +, ColE1 replicon	
pKNOCK-Km	Conjugative suicide vector Km^R^	Alexeyev, 1999
pBBRMCS-5	Cloning vector Gm^R^	Kovach et al., 1995
pKmgladI	*glad* I internal fragment cloned in pKNOCK-Km	This study
pLgladIR	pLAFR3 containing *B. gladioli* ICMP11096	This study
	Genomic DNA	
pBBgladIR	pBBRMCS-5 carrying a 3544 bp fragment containing *B. gladioli* quorum sensing genes	This study

### Recombinant DNA Techniques

Recombinant DNA techniques, including digestion with restriction enzymes, agarose gel electrophoresis, Southern blot analysis of restriction digested DNA, ligation with T4 DNA ligase, and transformation of *E. coli* were performed as previously described (Sambrook et al., 1989). Plasmids were purified by using EuroGold plasmid miniprep kit, and agarose gel electrophoresis purification of DNA fragments was performed with EuroGold gel purification kit (EuroClone, Italy). Genomic DNA was isolated by sarkosyl-pronase lysis. PCR amplifications were made using GoTaq DNA polymerase (Promega). DNA sequencing was performed by Macrogen Europe.

### Identification and Inactivation of Quorum Sensing

#### Cosmid Library

A cosmid library was constructed to identify the QS genes of WT *Bga* strain. More specifically, partial *Eco*RI digested genomic DNA from *Bga* strain ICMP11096 was cloned into the cosmid vector pLAFR3 and packaged into phage particles by using the Gigapack Gold packaging extract (Stratagene). The recombinant phage particles were transduced to *E. coli* HB101. The cosmid library was conjugated *en masse* into the *C. violaceum* CV026 AHL biosensor (McClean et al., 1997). One transconjugant which showed restoration of QS regulated violacein production, presumably containing the *Bga* QS genes, was further isolated to purify the pLgladIR cosmid. The pLgladIR cosmid was cut with different restriction enzymes and analyzed by Southern blot using the *tofI* gene from *Burkholderia glumae* as a probe (Devescovi et al., 2007). The *tofI* gene is coding for the acyl homoserine lactone synthase in *B. glumae* and it was chosen because of the high phylogenetic closeness of the two species. The *gladI* gene was localized on a *Xho*I fragment of approximately 3500 bp. This fragment was further cloned in pBBRMCS-5, to generate pBBgladIR. When conjugated in CV026, pBBgladIR was able to restore the violacein production. The plasmid was partially sequenced and the *glad-*I and a luxR homolog (*glad-*IR) were identified.

#### Construction of Glad I QS Mutants

To generate the knockout mutant of *gladI* gene, an internal fragment of *gladI* gene (261 bp) was PCR amplified by using the following primers generated in the current study gladI_Fw 5′-TGCGCGCGACTATTGCCGAC-3′ and gladI_Rev 5′-GAACAGCCGCTCGATACTGC-3′. The primers were chosen from the partially sequenced pBBgladIR. The fragment was then cloned in pGEM vector (Promega) sequenced and subsequently cloned as an *Eco*RI fragment in the pKNOCK-Km vector (Alexeyev, 1999) generating pKmgladI. This plasmid was then used as a suicide delivery system and conjugated in the WT *Bga* strain in order to obtain the genomic mutant ICMP11096*glad*-I. The fidelity of the marker exchange event was confirmed by PCR analysis. To complement the *glad-*I mutation, the plasmid pBBgladIR was transferred to ICMP11096*glad*-I by triparental mating, generating ICMP11096*glad*-IR.

### Antimicrobial Effect

The *N.*AHLs-deficient mutant (ICMP11096*glad*-I) and the complemented one (ICMP11096*glad*-IR) were evaluated for their ability to inhibit the growth of *B. megaterium* and *E. coli* in dual agar plate assay following the method of Lavermicocca et al. (1997) as reported by Camele et al. (2019). In particular, small masses of fresh bacterial cultures were transferred in the center of 90 mm Ø MMA Petri dishes. All plates were then sprayed with the single target bacterial suspension containing 10^8^ Colony Form Unit (CFU)/ml. The antagonistic activity was determined by measuring the diameter of inhibition zones and was expressed as the antagonistic capacity percentage. The test was repeated twice with three replicates.

### Hemolytic Effect

The hemolytic effect of the above mentioned two mutants was evaluated against the cell membrane of erythrocyte’s (RBCs) as explained by Sakr et al. (2018) following the method of Munsch and Alatossava (2002). The hemolysis ability was indicated by measuring the diameter of transparent area in comparison to WT strain as reported in the previous study by Elshafie et al. (2017a). The test was repeated twice with three replicates.

### Extracellular Hydrolytic Enzymes

The possible hydrolytic enzymes that might be produced by the two studied mutants of *Bga* was screened as specified by Elshafie et al. (2017a). In particular, chitinase and protease activities were determined according to Tahtamouni et al. (2006). Cellulase activity was detected following the method of Essghaier et al. (2009) and glucanase activity was detected according to the method of Teather and Wood (1982). Amylase, pectinase and polygalacturanase activities were detected following the methods of Sung et al. (1993) and Bhardwaj and Garg (2010). The enzymatic activity was evidenced by the appearance of clear halos around the colonies and their diameters were measured in (mm) compared to WT stain.

### Pathogenicity Assay

Pathogenicity test of WT and the two studied mutants of *Bga* was performed on *A. bisporus* flesh pseudo tissues as following: 20 μl drops of each bacterial suspensions at 10^6^, 10^4^, and 10^3^ CFU/ml were deposited on carpophores of *A. bisporus* and then were incubated for 96 h at 22 ± 25°C (Elshafie et al., 2017c). Carpophores treated with 20 μl drops of sterile distilled water were used as control. The virulence effect was observed after 4 days of incubation through the evaluation of the discoloration intensity. The virulence effect of WT *Bga* strain, obtained in the previous study by Elshafie et al. (2017a), was used here only for a comparison purposes.

### HPLC Fractionation

#### Purification of Bioactive Substances

The purified filtrate of ICMP11096*glad-*I mutant was analyzed by HPLC as reported previously by Elshafie et al. (2017c). Aliquots (30 mg.ml^–1^) of lyophilized bioactive metabolites derived from 5 day old bacterial culture filtrates were diluted in sterile distilled water. An aliquot (10 ml) of the prepared mixture, containing 300 mg of lyophilized substances, was loaded on a cartridge syringe (Strata C18-T) prewashed with 2 ml of methanol and 2 ml of distilled water. The cartridge was subsequently washed with 1 ml distilled water and later the bioactive substances were recovered by adding 1 ml of methanol 98%.

#### HPLC Fractionation

The methanol fractions were analyzed in HPLC-Agilent 1200 series austere following the specific analytical procedures described below. The separation was obtained with an Agilent ECLIPSE XDB, C18 (4.6 × 150 mm, 5 μm). The injected volume was 20 μl; column and autosampler chamber temperatures were at 25 and 4°C, respectively. Flow rate was 1 ml.min^–1^ and mobile phase were A: 0.2% formic acid (FA) in H_2_O and B: 0.1% FA in Acetonitrile (CH_3_CN) with the following gradients (A%: B%) 80–20 from 0 to 5 min; 60–40 from 5 to 60 min and 80–20 from 60 to 65 min. The HPLC chromatogram was obtained at a wave length λ = 380 nm.

### Statistical Analysis

The experimental outputs were statistically analyzed using statistical Package for the Social Sciences SPSS (version 13.0, Prentice Hall: Chicago, IL, United States, 2004). Experimental data was expressed as mean ± SD and comparisons were employed by a one way ANOVA followed by Tukey *post hoc* test for detecting any significance of the investigated data regarding the studied biological activities at *P* < *0.05*.

## Results and Discussion

### Antimicrobial Activity

Results of ICMP11096*glad*-I mutant showed significant reduction of antimicrobial activity in MMA substrate in comparison with the WT strain (Table 2) and this reduction could be due to the absence of signal molecules *N.*AHLs which indicate their regulatory role in the QS process enhancing the production of bioactive secondary metabolites. On the other hand, ICMP11096*glad*-I complemented mutant explicated similar values of antimicrobial activity of the respective WT strain which indicates that it was able to regain its bioactivity (Table 2).

**TABLE 2:** Antimicrobial, hemolytic, and enzymatic activities of wild type (WT), ICMP11096*glad*-I and ICMP11096*glad*-IR mutants of *B. gladioli* pv. *agaricicola*.

**Studied strains**	**Antimicrobial effect^a^**		**Erycrocyte cell lysis^b^**	**Extracellular hydrolytic enzymes^c^**						
			
	***B. meg***	***E. col***		**Protease**	**Cellulase**	**Chitinase**	**Amylase**	**Glucanase**	**Pectinase**	**Polygalacturonase**
Bga WT	35 ± 1.5	30 ± 2.7	15 ± 2.1	12 ± 1.3	–	25 ± 3.6	–	28 ± 3.5	–	–
Bga *gladI*	8 ± 0.7	5 ± 0.2	–	5 ± 0.4	–	5 ± 0.7	–	12 ± 2.1	–	–
*Bga gladIR* complemented	33 ± 2.4	28 ± 3.3	12 ± 1.5	9 ± 1.9	–	12 ± 1.7	–	22 ± 2.8	–	–

*^*a*^Antimicrobial effect of wild type (WT) and mutants of *Bga* ICMP11096 strain against *Bacillus megaterium* (*B. meg*) and *E. coli* (*E. col*) expressed as antagonistic capacity percentage; ^*b*^Hemolytic effect against erythrocyte cell membrane of fresh bovine blood; ^*c*^Extracellular hydrolytic enzymes (hydrolysis zones in mm) caused by WT, ICMP11096*glad*-I and ICMP11096*glad*-IR mutants.*

### Hemolytic Activity

Results of observations of ICMP11096*glad*-I mutant, expressed as average diameters of hemolysis zones (mm), did not show any hemolytic activity compared to WT strain, which has been investigated in the previous study, ranged between 31.0 ± 6.5 and 75.0 ± 11.5 mm (Elshafie et al., 2017a). The ICMP11096*glad*-IR complemented mutant was able to regain the majority of hemolytic effect level close to the respective WT *Bga* strain (Table 2). These outcomes indicated that *N.*AHLs plays the principal role in production or/and regulation of hemolysis factors in agreement with the hypothesis of Munsch and Alatossava (2002) who stated that several *Pseudomonas* and other related bacteria associated with the cultivated mushrooms *A. bisporus*, such as *Burkholderia* sp., are hemolytic.

### Extracellular Hydrolytic Enzymes

The ICMP11096*glad*-I mutant showed a significant reduction of enzymatic activity especially with protease, chitinase and glucanase enzymes (Table 2). There is no production of cellulase, amylase, pectinase, and polygalacturonase. Furthermore, the reduction of proteolytic, chitinolytic, and glucanolytic activities could contribute to lowering the virulence effect of the mutant strain against *A. bisporus* tissues in agreement with Ordentlich et al. (1988). Furthermore, ICMP11096*glad*-IR complemented mutant showed closed values of hydrolysis effect to the WT strain of *Bga* (Table 2).

### Pathogenicity Assay

**FIGURE 1:**
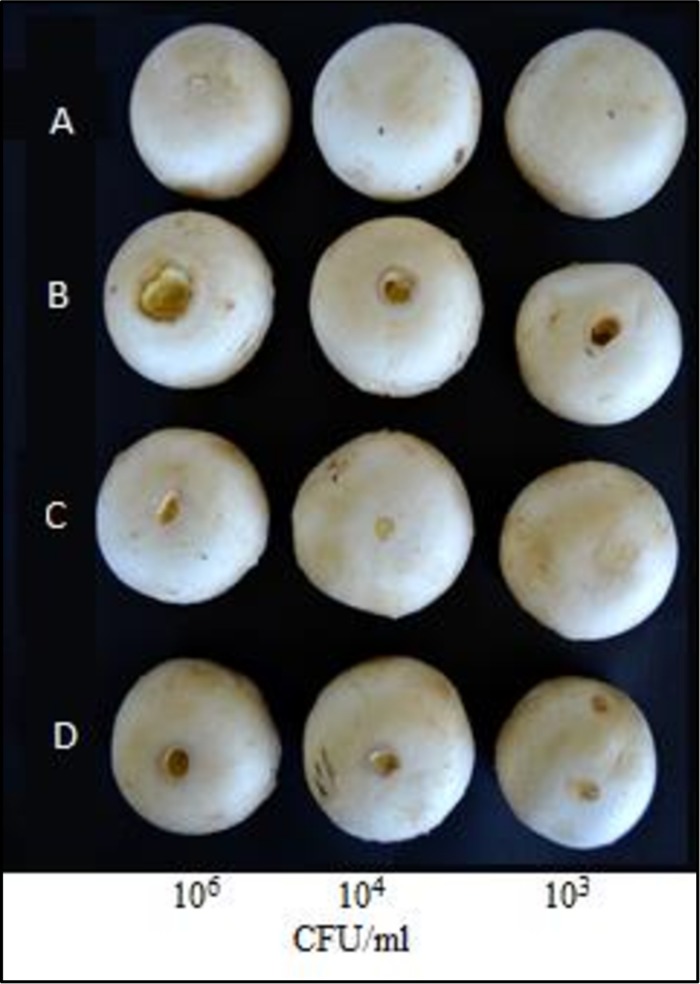
Pathogenicity assay on carpophores of *Agaricus bisporus*. A = control (sterile distilled water); B = wild type (WT) strain ICMP 11096; C = *N*.AHLs-deficient mutant (*ICMP11096glad*-I); D = complemented plasmid mutant (*ICMP11096glad*-IR); 10^6^, 10^4^, and 10^3^ CFU/ml are the tested concentration of bacterial suspensions.

The ICMP11096*glad*-I mutant strain did not show any color change of the artificially infected *A. bisporus* tissues. The ICMP11096*glad*-IR complemented mutant was able to regain the virulence effect similar to WT strain (Figure 1). This result indicates that *N.*AHLs signal molecule is involved in the regulation of virulence factors which might be related also to the hydrolytic enzymes in accordance with Chowdhury and Heinemann (2006).

### HPLC Analysis

**FIGURE 2:**
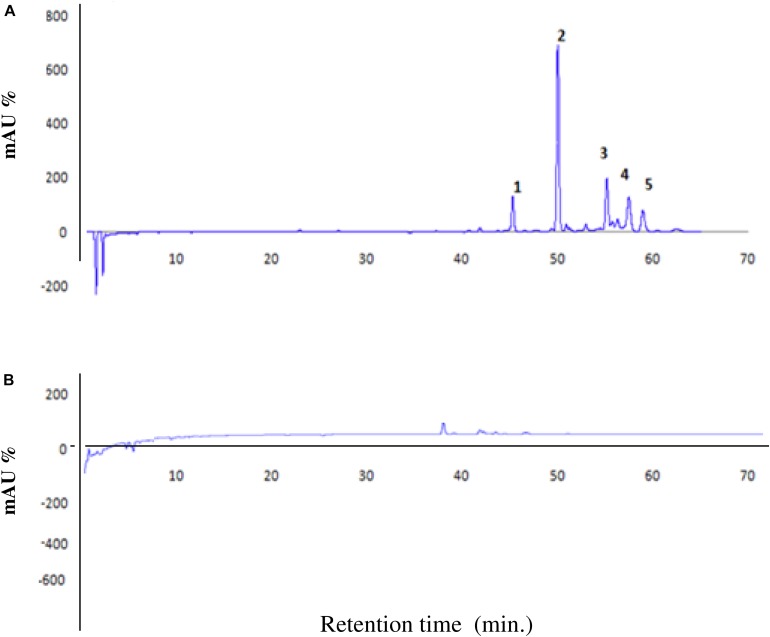
High performance liquid chromatography (HPLC) chromatogram of WT strain *Bga* ICMP11096 **(A)** and *N*.AHLs-deficient mutant (*ICMP11096glad*-I) at 380 nm **(B)**.

The HPLC chromatographic analysis of *N.*AHLs-deficient mutant *glad* I demonstrated a highly reduction of the five principal peaks typically present in WT strain from quantitative point of view (Elshafie et al., 2017c; Figure 2). The absence or trace level of the fractions is also in agreement with the above results of the pathogenicity assay which assumed that the lack of bioactive compounds in mutant strain might be linked to the absence of virulence factors.

## Conclusion

As previously described by Zhou et al. (2003) in *Burkholderia ambifaria* BC-F, a biocontrol strain reported previously to exhibit broad-spectrum antifungal activity, the inactivation of the QS genes *N*.AHL synthase (bafI) and *N*.AHL-binding transcriptional activator (bafR) leads to a decreased antifungal activity. Similarly, the inactivation of the AHL synthase (gladI) in *Bga* caused the loss of the antibacterial activity and other phenotypic traits. The *N.*AHLs-deficient mutant showed a high reduction of the antimicrobial activity, complete absence of hemolytic effect and a significant reduction of hydrolytic enzymes (chitinase, protease, and glucanase). It is also concluded that there are various factors for regulating the genes implicated in the production of antimicrobial substances different from those responsible for the hemolytic substances. The above mentioned hydrolytic enzymes in WT strain could be strongly implicated in soft rot disease of *A. bisporus*. The outcomes from the current study are promising in controlling mushroom soft rot disease and clarifying the regulatory role of *N.*AHLs-mediated QS in biological traits of *Bga* are mostly likely important in the pathogen virulence.

## Data Availability Statement

The raw data supporting the conclusions of this article will be made available by the authors, without undue reservation, to any qualified researcher.

## Author Contributions

HE and SB: conceptualization. GD and VV: DNA techniques. SB and IC: data curation and review the manuscript. HE, IC, and GD: writing the original draft and editing.

## Conflict of Interest

The authors declare that the research was conducted in the absence of any commercial or financial relationships that could be construed as a potential conflict of interest.
